# From regeneration to osteoarthritis in the knee joint: The role shift of cartilage-derived progenitor cells

**DOI:** 10.3389/fcell.2022.1010818

**Published:** 2022-10-20

**Authors:** Wenguang Liu, Meng Feng, Peng Xu

**Affiliations:** ^1^ Honghui Hospital, Xi’an Jiaotong University, Xi’an, China; ^2^ Department of Orthopedics, The Second Affiliated Hospital of Xi’an Jiaotong University, Xi’an, China

**Keywords:** cartilage-derived progenitor cell, role shift, osteoarthritis, regeneration, hallmarks

## Abstract

A mount of growing evidence has proven that cartilage-derived progenitor cells (CPCs) harbor strong proliferation, migration, andmultiple differentiation potentials over the past 2 decades. CPCs in the stage of immature tissue play an important role in cartilage development process and injured cartilage repair in the young and active people. However, during maturation and aging, cartilage defects cannot be completely repaired by CPCs *in vivo*. Recently, tissue engineering has revealed that repaired cartilage defects with sufficient stem cell resources under good condition and bioactive scaffolds *in vitro* and *in vivo*. Chronic inflammation in the knee joint limit the proliferation and chondrogenesis abilities of CPCs, which further hampered cartilage healing and regeneration. Neocartilage formation was observed in the varus deformity of osteoarthritis (OA) patients treated with offloading technologies, which raises the possibility that organisms could rebuild cartilage structures spontaneously. In addition, nutritionmetabolismdysregulation, including glucose and free fatty acid dysregulation, could influence both chondrogenesis and cartilage formation. There are a few reviews about the advantages of CPCs for cartilage repair, but few focused on the reasons why CPCs could not repair the cartilage as they do in immature status. A wide spectrum of CPCs was generated by different techniques and exhibited substantial differences. We recently reported that CPCs maybe are as internal inflammation sources during cartilage inflammaging. In this review, we further streamlined the changes of CPCs from immature development to maturation and from healthy status to OA advancement. The key words including “cartilage derived stem cells”, “cartilage progenitor cells”, “chondroprogenitor cells”, “chondroprogenitors” were set for latest literature searching in PubMed and Web of Science. The articles were then screened through titles, abstracts, and the full texts in sequence. The internal environment including long-term inflammation, extendedmechanical loading, and nutritional elements intake and external deleterious factors were summarized. Taken together, these results provide a comprehensive understanding of the underlying mechanism of CPC proliferation and differentiation during development, maturation, aging, injury, and cartilage regeneration *in vivo*.

## Introduction

Stem cells exhibit strong self-renew and multiply differentiation abilities, which lay the foundation of application to musculoskeletal diseases (Mousaei Ghasroldasht et al., 2022). Till now, several types of stem cells were isolated and generated, including mesenchymal stem cells (MSCs), hematopoietic stem cells (HSCs), embryonic stem cells (ESCs) and induced pluripotent stem cells (iPSCs). Each of them showed significant advantages and disadvantages. ESCs harbored strongest pluripotency but with teratomas risk (Castro et al., 2013). iPSCs were artificially made by reprogramming adult somatic cells using specific factors. They share similar characteristics of ESCs, but were still not investigated enough (Sharkis et al., 2012). Hematopoietic stem cells (HSCs) and mesenchymal stem cells (MSCs) are with limited multiply differentiation properties. HSCs are the only types of cell approved by the Food and Drug Administration (FDA) for stem cell-based therapy, but they still may cause transplant rejection (Müller et al., 2016). MSCs could mainly differentiate toward mesoderm cells, including bone, cartilage, fat and muscle. Meanwhile, MSCs have been isolated from these tissues, which could harbor different preference of differentiation. For example, adipose-derived stem cells (ADSC) have a strong adipogenic differentiation ability (Bourin et al., 2013). MSCs showed lower MHC I and no MHC II expression, which were though as low immunogenic potential. However, recent studies proved that MSCs were not “immune privileged” and could induce immunological response as well (Ankrum et al., 2014). Therefore, how to decrease immunological reflect and increase specific differentiation characteristics were needed to be considered for stem-cell based therapy. Autologous and tissue specific stem cells may be an underlying solution.

The nature of hyaline cartilage is an avascular tissue with limited self-healing properties. Once injury or degeneration appears to overextend the tissue’s self-renewal capacity, long-term and low-level inflammation exists in the joint, which contributes to osteoarthritis onset and OA-related repair. Although inflammatory reaction is a process part of the repair initiation, long-lasting inflammatory conditions hinder cartilage repair and cause flare-up of OA symptoms. In many other tissues, tissue-specific stem cells can contribute to tissue regeneration. However, the full-thickness of cartilage could not be fully repaired spontaneously (Jiang and Tuan, 2015). Cartilage-derived progenitor cells (CPCs) are identified as a promising cell subpopulation with strong proliferation and chondrogenic potential for cartilage regeneration *in situ.* However, cartialge defects actually occur in elderly individuals and even in healthy middle-aged people (Cicuttini et al., 2005). In symptomatic OA patients, cartilage defects tend to progress during 2 years of follow-up (Davies-Tuck et al., 2008). It is possible that CPCs failed to repair the degenerated cartilage and reverse the OA cartilage pathogenesis aggravation. On the other hand, CPCs were successfully used for cartilage repair with amplification culture and the combination of scaffolds (Rikkers et al., 2022). Therefore, the local environment of cartilage or joints may be the stumbling barrier for CPC proliferation and chondrogenesis, which are the key factors for cartilage regeneration.

**FIGURE 1:**
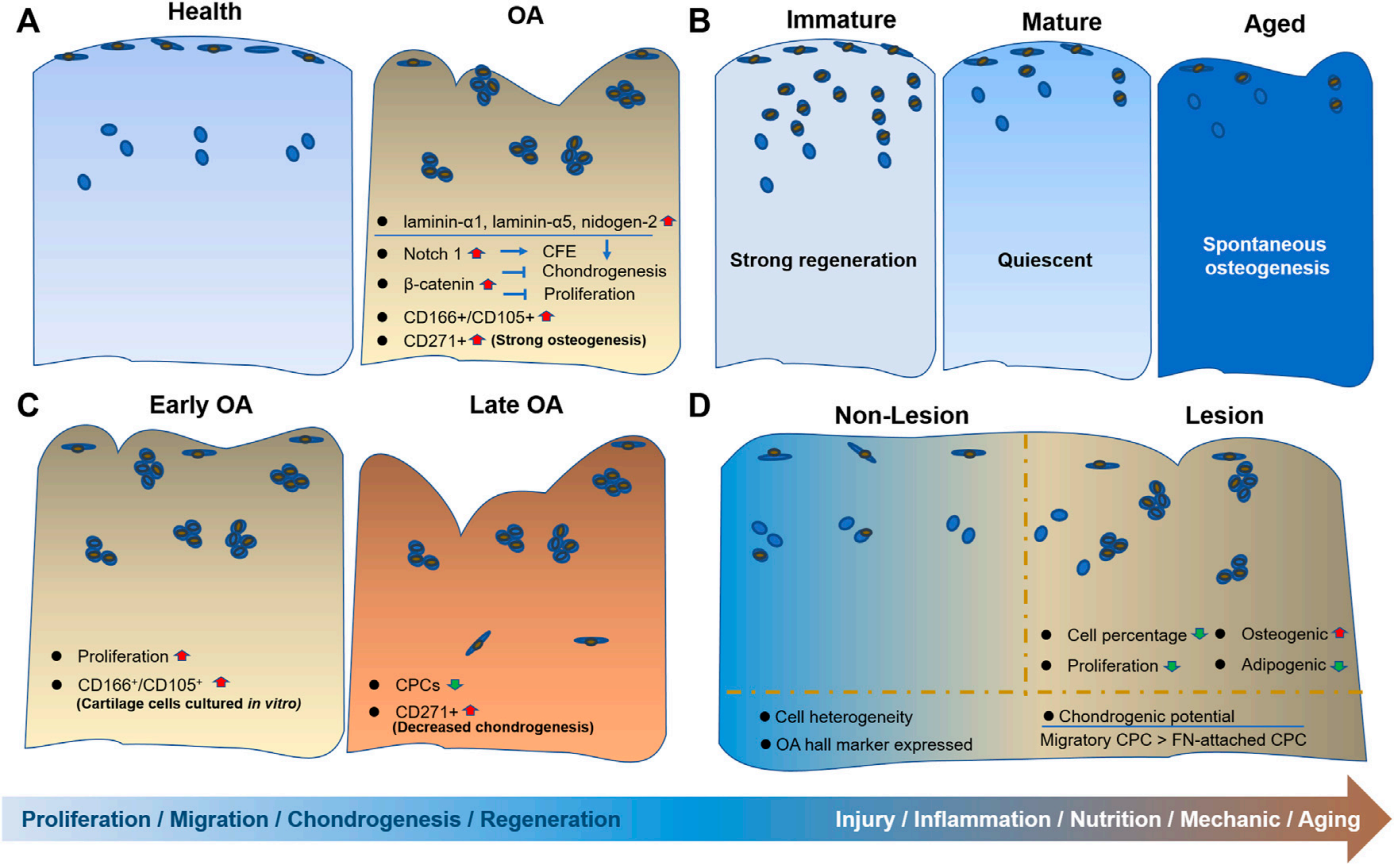
The alterations of CPCs during aging and disease progression. Schematic overview of CPC alterations is depicted in different stages of cartilage, including **(A)** from healthy to OA cartilage; **(B)** from immature to aged cartilage; **(C)** from early OA to late OA cartilage; and **(D)** the comparison between nonlesion and lesion cartilage.

In this review, we summarized the internal and external changes of CPCs from regeneration status to degeneration and disease status (Figure 1). The characteristics of origins of CPCs, from immature to mature tissue, including their differentiation potential and injury response capacity. In addition, dedifferentiated chondrocytes showed similar properties to CPCs, which were also included. Furthermore, the alterations in CPCs caused by OA were summarized. The inner and outer environment, including mechanical loading, nutrition supply, inflammation and aging, was given special attention.

## Characteristics of CPCs

**FIGURE 2:**
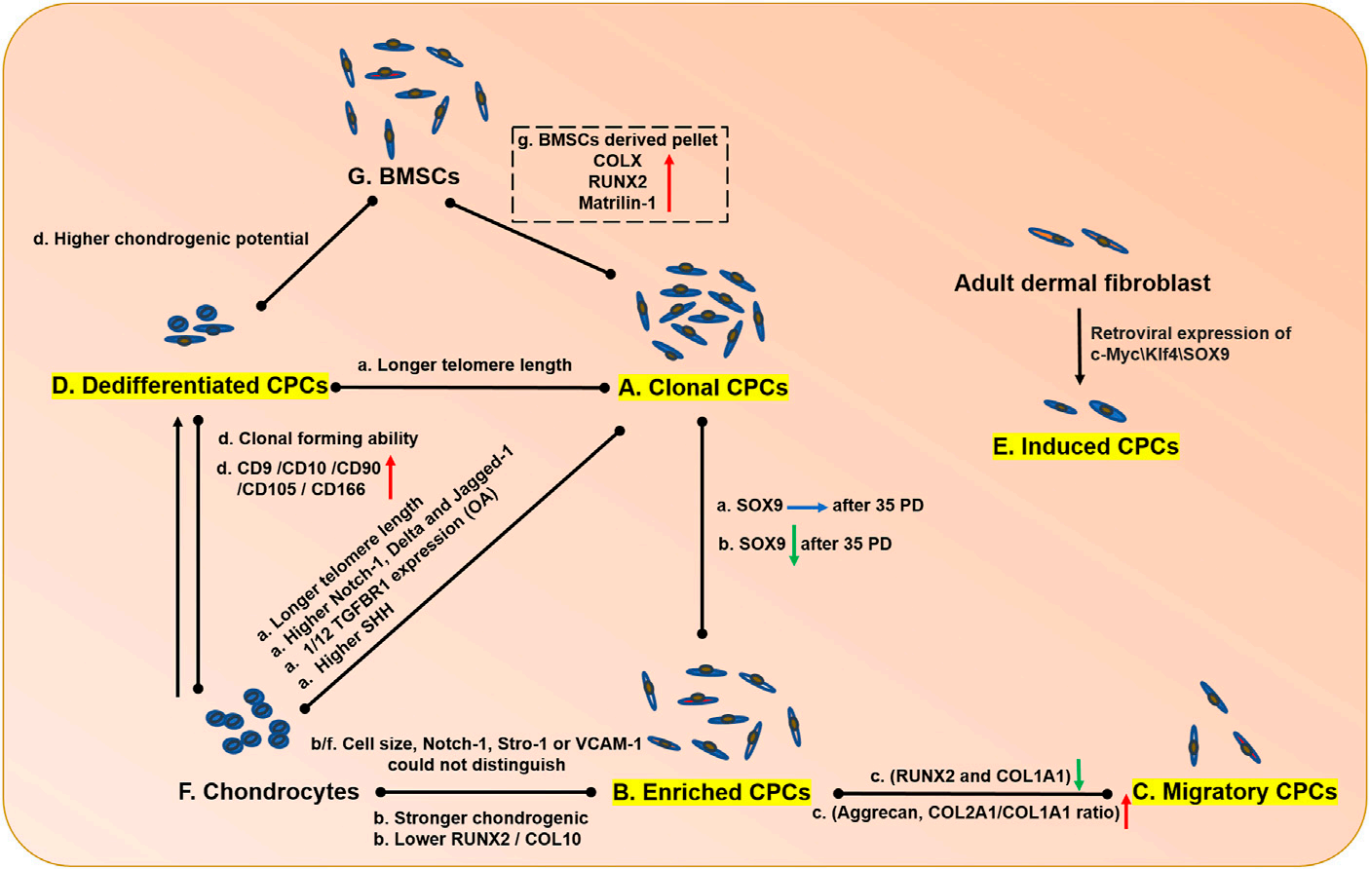
The comparison of CPCs from different sources, chondrocytes and BMSCs. The CPCs isolated from different methods exhibited different properties. The capitalized **(A–G)** means single cell derived clonal CPCs, enriched CPCs, migrated CPCs from cartilage tissue, dedifferentiated CPCs derived from chondrocytes and specific factors induced CPCs, chondrocytes and BMSC, respectively. The capacities of proliferation differentiation, and migration were compared. a, b, c, d, e, f, g in lowercase means the cell properties in A, B, C, D, E, F, G cells compared with cells being connected by line, respectively. The red arrow means upregulation; the green arrow means downregulation; the blue arrow means unchanged; the black arrows mean cell transition.

Chondrocytes were thought to be the only type of cell in cartilage for a long time. CPCs were first-time identified and isolated in the superficial cartilage of a 7-day born bovine via the fibronectin attachment method in 2004 (Dowthwaite et al., 2004). Fibronectin-attached cells showed lower RUNX2 and COLX expression than nonfibronectin-attached cells and unselected cartilage-derived cells (Vinod et al., 2020a) (Figures 2B vs. F). Several markers were used as single or combined markers for CPCs characterization, such as CD49e, CD73, CD90, CD105, CD106, CD146, CD166, Notch 1, STRO-1 and smooth-muscle actin (Jiang and Tuan, 2015). Some of those markers are overlapped with BMSCs, including CD73, CD105 and STRO-1 (Jayasuriya and Chen, 2015). To date, there are no precise and specific biomarkers for the cartilage-resident progenitor cells. Small elongated morphology and NOTCH 1 had ever been reported to distinguish CPCs from other cell groups. Although the usage of NOTCH 1 failed to distinguish cell differentiation potential, NOTCH 1 significantly affected the colony-forming ability (CFE) of CPCs (Nelson et al., 2014). Therefore, neither NOTCH 1 nor cell size could sort the cell types with different differentiation potential from adult bovine cartilage (Karlsson et al., 2008). Recently, the combination of a couple of cellular differentiation (CD) markers were used for CPC identification. Interestingly, CD166^-^ cells showed no chondrogenic capacity (Pretzel et al., 2011), but CD166^low/−^CD73^+^CD146^low/−^LIN^−^CD44^low^ cells can only undergo chondrogenesis (Wu et al., 2013).

## Cell heterogeneity in cartilage

CPCs were discovered topographically as mesenchymal stem-like cells, which are heterogeneous. Evidence showed that the CPCs had different chondrogenic/osteogenic potentials (Nelson et al., 2014), growth kinetics, telomere lengths, and senescence indices (Fellows et al., 2017). In non-lesion areas of one OA patient, human CPCs (hCPCs) showed heterogeneity with different differentiation potentials, but both exhibited OA hallmarks (Jayasuriya et al., 2018). In the different gender (Koelling and Miosge, 2010) and the distribution in the full-thickness of cartilage, CPCs exhibited different trends during differentiation. For example, CPCs in the deep zone harbor more chondrogenic and osteogenic potential than superficial CPCs (Yu et al., 2014). Because of the heterogeneity in cartilage, Vinod et al. aimed to mimic the mixture condition in cartilage with different percentages of chondrocytes and CPCs, but they did not obtain positive results of chondrogenesis (Vinod et al., 2019a). In 2019, signal cell RNA sequence technology further proved that OA cartilage contained seven types of cells (Ji et al., 2019), which exhibited complexity of the exact cell components in cartilage. More studies should focus on the function of each type of cell, especially the multiply functional CPCs. Up to date, fibronectin enriched CPCs, single cell derived clonal CPCs, migrated CPCs from cartilage tissue, dedifferentiated CPCs derived from chondrocytes and specific factors induced CPCs have been isolated and generated to be investigated (Figure 2 and Table 1). Although they were all thought as CPCs, they exhibited obvious differences in comparison.

**TABLE 1:** The comparison of CPCs from different sources, chondrocytes and BMSCs.

Species	Age	Gender	Sites	Severity	Isolated method	Major results	Ref
Human	Average age: 60.1 years	6 M/9 F	Femoral condyle cartilage	Unaffected areas of OA cartilage	NGF stimulated migration	CPCs were activated by IL1b and NGF signaling in OA.	Jiang and Tuan (2015)
	N = 3			Normal cartilage			
	N = 3			Fetal cartilage			
Mouse	6 weeks old	Male	Knee articular cartilage	Normal cartilage	Fibronectin attachment	Fibronectin enhanced the proliferation, migration and chondrogenic differentiation capacity of CPCs via the integrin α5β1 pathway	Tao et al. (2018)
Rat	8 weeks old		Articular cartilage	Normal cartilage	Colonies formation	Leptin decreased CPCs migration and chondrogenic potential, meanwhile increased osteogenic potential	Zhao et al. (2016)
Horse	2–8 years old (N = 5)		Distal end of metacarpal bone III	Normal cartilage	Fibronectin attachment	Pellets derived from BMSC expressed COLX, RUNX2 and Matrilin-1, whereas the pellets from CPCs did not	McCarthy et al. (2012)
Human	47–71 years old (N = 51)		Knee articular cartilage	Normal looking OA cartilage	Dedifferentiated chondrocyte	Dedifferentiated chondrocytes showed similar properties as BMSC but showed higher chondrogenic potential, which could be used for cartilage repair	Jiang et al. (2016)
Human	56–68 years (N = 3)	Female	Knee articular cartilage	Normal looking OA cartilage	Fibronectin attachment	OA-CPC showed 1/12 TGFBR1 compared with OAC, resulting different effect of TGFb on OA-CPC and OAC.	Liu et al. (2020b)
Human	18 years (N = 1)	Female	Cell line	Normal cartilage	Fibronectin attachment	OA-CPC expressed high SHH that could induce OA-CPC proliferation, chondrogenesis, hypertrophy, and replicative senescence and could suppress COL2A1, stimulate MMP13, and induces apoptosis in OAC.	Feng et al. (2021)
	68 ± 1.6 years (N = 18)	13 M/5 F	Cell lines and primary CPCs from normal looking knee articular cartilage	Normal looking OA cartilage			
Human	32–89 years old (N = 18)		Lateral femoral condyles cartilage	Normal cartilage	A mixture of chondrocyte and CPCs	CD10, CD90, CD105, CD166 were upregulated in OA chondrocyte during monolayer culture	Diaz-Romero et al. (2005)
Human	53.67 ± 5.9 years (N = 3)	1 M/2 F	Knee articular cartilage	Grade 4 OA cartilage	Fibronectin attachment	Fibronectin attached CPCs exhibited lower levels of hypertrophy markers (RUNX2 and COL10A1) compared with non-attached cells and total cells in OA cartilage	Kachroo et al. (2020)
Bovine	7 days		Metacarpophalangeal joints	Normal cartilage	Fibronectin attachment	Clonal CPCs could maintain telomerase activity and Sox9 expression after long time culture	Khan et al. (2009)
Human	10–57 years old (N = 9)		Femur condyle cartilage	Normal cartilage	Fibronectin attachment	Clonal CPCs showed longer telomere length and stronger telomerase activity than chondrocyte	Williams et al. (2010)
Caprine	Young and mature (N = 3)	Female	Lateral femoral condyle cartilage	Cartilage defect		CPCs combined with type I/III collagen membrane were used for cartilage defect	
Bovine			Lateral tibial plateau cartilage	4-mm-diameter defects with 2-mm depth	*Ex vivo*	Short-term enzymatic treatment could activate CPCs migration, which may benefit cartilage repair	Schminke and Miosge (2014)
Human	65–75 years old		Adjacent to the main defect	OA cartilage	Migration	CPCs in late OA showed strong migration capacity. Knockdown RUNX2 could enhance SOX9 and aggrecan	Koelling et al. (2009)
Human	55 ± 4 years (N = 3)		Knee articular cartilage	Grade 4 OA cartilage	Migration or fibronectin attachment	Migratory CPC: lower levels of hypertrophy markers (RUNX2 and COL1A1), higher levels of chondrogenic markers (Aggrecan and COL2A1/COL1A1 ratio)	Vinod et al. (2021)
Bovine	15–24 months old		Tibial plateau cartilage	Normal cartilage	Blunt impact or scratch stimulated migration	CPCs were more sensitive to chemotaxis, cell lysates, condition medium and HMGB-1 than chondrocytes	Seol et al. (2012)
Human	29, 34, and 46 years old (N = 3)	Male					
Human	45–87 years old (N = 71)		Knee articular cartilage	Non-fibrillated regions	Migration	PDGF or IGF-1 stimulated CPCs migration, which could be abolished by IL-1b or TNFa but not IL-6	Joos et al. (2013)
Bovine			Tibial plateaus	Normal cartilage	Migration	CPCs showed phagocytic capacity when injury happened	Zhou et al. (2016)

### Enriched CPCs and clonal CPCs

Fibronectin attached method was the most common method for CPC isolation. Single cell derived and expanded colony with more than 32 cells were thought as clonal CPCs. Fibronectin itself (via integrin ɑ5β1 receptor) benefited enriched CPCs proliferation, migration and chondrogenesis (Tao et al., 2018). Similar to BMSC, BMP2 and leptin could induce cell hypertrophy and osteogenesis (Zhao et al., 2016). In addition to these similarities, several genes and chondrogenic potential were quite different compared with BMSCs, which may prove that local CPCs are more suitable for cartilage repair. For example, equine CPCs (eCPCs) showed no collagen type X expression and lower runx2 and matrilin-1 expression under pellet culture condition (Figures 2A vs. G) (McCarthy et al., 2012).

Compared with enriched CPCs, only clonal CPCs, could maintain SOX9 expression after 35 passages (Figures 2A vs. B) (Jiang et al., 2016), which suggest that enriched CPCs were still heterogeneous compared with clonal CPCs. CPCs and chondrocytes are the two main types of cells in cartilage. Previous studies often showed several controversial functions in cartilage with same factors, such as TGFβ. Between clonal CPCs and chondrocytes (Figures 2A vs. F), we previously proved that clonal CPCs were with only 1/12 TGFR1 expression of chondrocyte, which caused the opposite functions in cartilage (Liu et al., 2020b). Similarly, Sonic Hedgehog (SHH) inhibited COL2A1 expression and induced degeneration in chondrocyte. On the contrary, it could induce clonal CPCs proliferation and chondrogenesis, but it also induced hypertrophy and senescence of clonal CPCs (Feng et al., 2021).

### Dedifferentiated CPCs

OA chondrocyte (OAC) loses its phonotype after several passages *in vitro*. However, it dedifferentiates toward fibroblast-like cells with upregulation of several CPC markers (CD10, CD90, CD105, CD166) (Figures 2D vs. F) (Diaz-Romero et al., 2005). Even compared with BMSC, they still exhibited higher chondrogenic potential (Figures 2D vs. G) (Jiang et al., 2016). CD49e is a CPC marker and CD49e-cells can become CD49e^+^ cells regardless of whether the adherent or nonadherent culture method is used (Kachroo et al., 2020). Williams et al. performed FACS analysis with passage five full-depth chondrocytes, in which CD105 (endoglin), CD166 (ALCAM), CD44 and CD29 (β1 integrin) were all more than 95% positive, which is consistent with our results. Our data showed that the number of CD166-positive cells increased from 2.9% to more than 95% after five passages (Liu et al., 2021). Furthermore, passaged chondrocytes exhibited senescent phenotypes, including a larger irregular morphology and an increase of secreted cytokines. Although differentiated CPCs showed similar surface markers and trilineage differentiation potential (Vinod et al., 2020a), but they have lower telomerase activity (Khan et al., 2009; Williams et al., 2010). Dedifferentiated OACs from different grades of OA cartilage showed no difference in cell markers or differentiation potential (Bernstein et al., 2013), which was quite different from CPCs. CPCs but not OACs may be influenced easily by OA progression.

#### Migratory CPCs

Migratory CPCs were reported in a cartilage explant model *ex vivo* due to cell migration from the explant. CPCs exhibit strong migration ability, which is essential for tissue repair (Schminke and Miosge, 2014). Even in late OA tissue, CPCs can migrate into OA cartilage approximately 1,200–1,400 µm deep after 2 days (Koelling et al., 2009). Compared with enriched CPCs, migratory CPCs had lower osteogenic markers and higher chondrogenic markers (Figures 2B vs. C) (Vinod et al., 2021). Superficial zone chondrocytes express high levels of α-smooth muscle actin, which is associated with cell migration (Seol et al., 2014). Migrating CPCs showed an elongated morphology with higher IL-6 and PRG4 expression and lower levels of cartilage extracellular matrix (ECM) genes, such as collagen type II and aggrecan (Seol et al., 2012). That is, ECM-related genes are negative for CPC migration. Several factors are involved in CPC migration, including environmental stimulation and migration-related gene expression. PDGF, IGF, high mobility group protein 1 (HMGB1), and supernatant with/without trauma all induced CPC migration, while inflammatory factors such as IL-1β and TNF-α abolished the migration effect. (Jiang and Tuan, 2015; Joos et al., 2013) (Figure 2C). CD44 and Runx2 expression could promote BMSC migration, while aging and maturation are both negative regulators of MSC migration. In the contrary, downregulation of Runx2 in CPCs does not influence the migration capacity but increases sox9 expression in 3D culture (Corradetti et al., 2017; Fujita et al., 2004; Koelling et al., 2009). Based on these results, CPCs, even late OA CPCs, harbored migration capacity responding to injury but failing to restore the cartilage defects in mature organisms. Besides, bCPCs were reported as macrophage-like cells (CD68 upregulated) in injured cartilage (Zhou et al., 2016), which showed another function of CPCs.

## CPCs in immature, mature and aged cartilage

**FIGURE 3:**
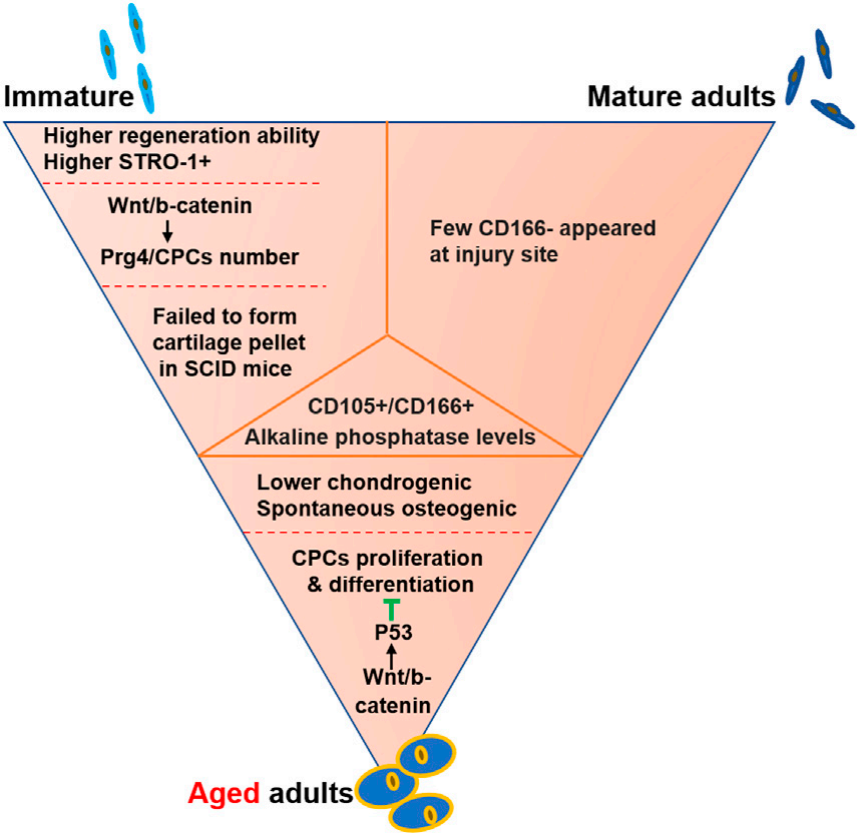
CPCs in immature, mature and elder cartilage. The common properties were listed in the middle orange triangle. Meanwhile, the main differences were showed near the sketch cells, including immature, mature and aged CPCs.

OA is an age-related degenerative disease that commonly happens in elder people. One possible reason is that immature cartilage defect could be easily repaired, but cartilage injury rarely heal itself when it comes to the mature organ (Table 2). The CPCs in immature, mature, aged cartilage shared some characteristics such as CD105/CD166 positive and alkaline phosphatase activity, but also showed different properties (Figure 3). Spontaneous repair occurred after injury made by knife in immature rats but not in mature rats, but there were few CD166^+^ cell at the injury site in mature rat (Mukoyama et al., 2015). Proteoglycan 4 (Prg4), a CPCs marker, marked different cell population at different time points. When it was labeled at E17.5, cells located in the superficial layer which acted as progenitors to the entire cartilage development. However, when it was labeled at 1-month-old, cells could consist only two-thirds upper of the articular cartilage (Kozhemyakina et al., 2015; Li et al., 2017). STRO-1, another factor used for CPCs, showed more positive cells were throughout the cartilage in 2-week-old (immature) mouse explants, while the positive cells gathered around the superficial zone with a decreased number in 3-month-old (mature) mouse explants (Otsuki et al., 2010). With the decrease in the STRO-1^+^ cell number, cartilage repair function is lost (Otsuki et al., 2010). The immature CPCs, such as fetal CPCs, were with stronger chondrogenic potential and utilized for cartilage repair successfully (Choi et al., 2016; Park et al., 2020). On the contrary, CPCs in aged adults exhibited lower chondrogenic and spontaneous osteogenic potentials (Chang et al., 2011). These different differentiation potentials may be caused by the different function of Wnt/β-catenin signaling, which benefited CPCs proliferation in immature tissue (Yasuhara et al., 2011) but inhibited proliferation and differentiation of CPCs (Xu et al., 2014). The underlying reasons of this contrary function may be caused by the basal level of Wnt/β-catenin signaling and should be investigated further.

**TABLE 2:** CPCs in immature, mature and elder cartilage.

Species	Age	Gender	Sites	Severity	Isolated method	Results	Ref
Rat	3 weeks old		Weightbearing region of the medial femoral condyles cartilage	Partial thickness articular cartilage injuries	*In vivo and ex vivo*	After injury, CD105+ and CD166+ cells were identified in the superficial and transitional zones of the articular cartilage, but few CD166+ cells were found in mature articular cartilage. No differences were found in mature and immature *ex vivo*	Mukoyama et al. (2015)
	12 weeks old						
Bovine	6–9 months		Distal femoral condyles cartilage	Normal cartilage	*Ex vivo*	Immature cartilage tissue harbored more STRO-1+ cells	Otsuki et al. (2010)
	>2 years						
Mouse	2 weeks				Injury induction	Immature cartilage tissue showed higher repair potential	
	3 months						
Human	20–24 weeks	3 M/5 F	Femoral condyle cartilage	Normal cartilage	CD105+/CD166+ sorting	Lower chondrogenic and spontaneous osteogenic differentiation were detected only in elder person	Chang et al. (2011)
	28–45 years	7 M/4 F					
	60–75 years	5 M/3 F					
Mouse	P3-P5		Epiphyseal articular cartilage	Normal cartilage	Fibronectin attachment	b-catenin signaling increased the number of CPCs and *prg4* expression in CPCs	Yasuhara et al. (2011)
Human	58 ± 65.2 years old (N = 5)	4 M/1 F	Femoral head cartilage	Normal cartilage	CD105+/CD166+ sorting	CPCs in OA showed decreased differentiation abilities and enhanced Wnt/b-catenin activity. Inhibition of Wnt/b-catenin signaling or activation this pathway by p53 could promote OA CPCs or normal CPCs proliferation and differentiation, respectively	Xu et al. (2014)
	49.5–55.5 years old (N = 10)	8 M/2 F	Femoral condyles cartilage	OA cartilage			

## CPCs alteration during OA progression

**FIGURE 4:**
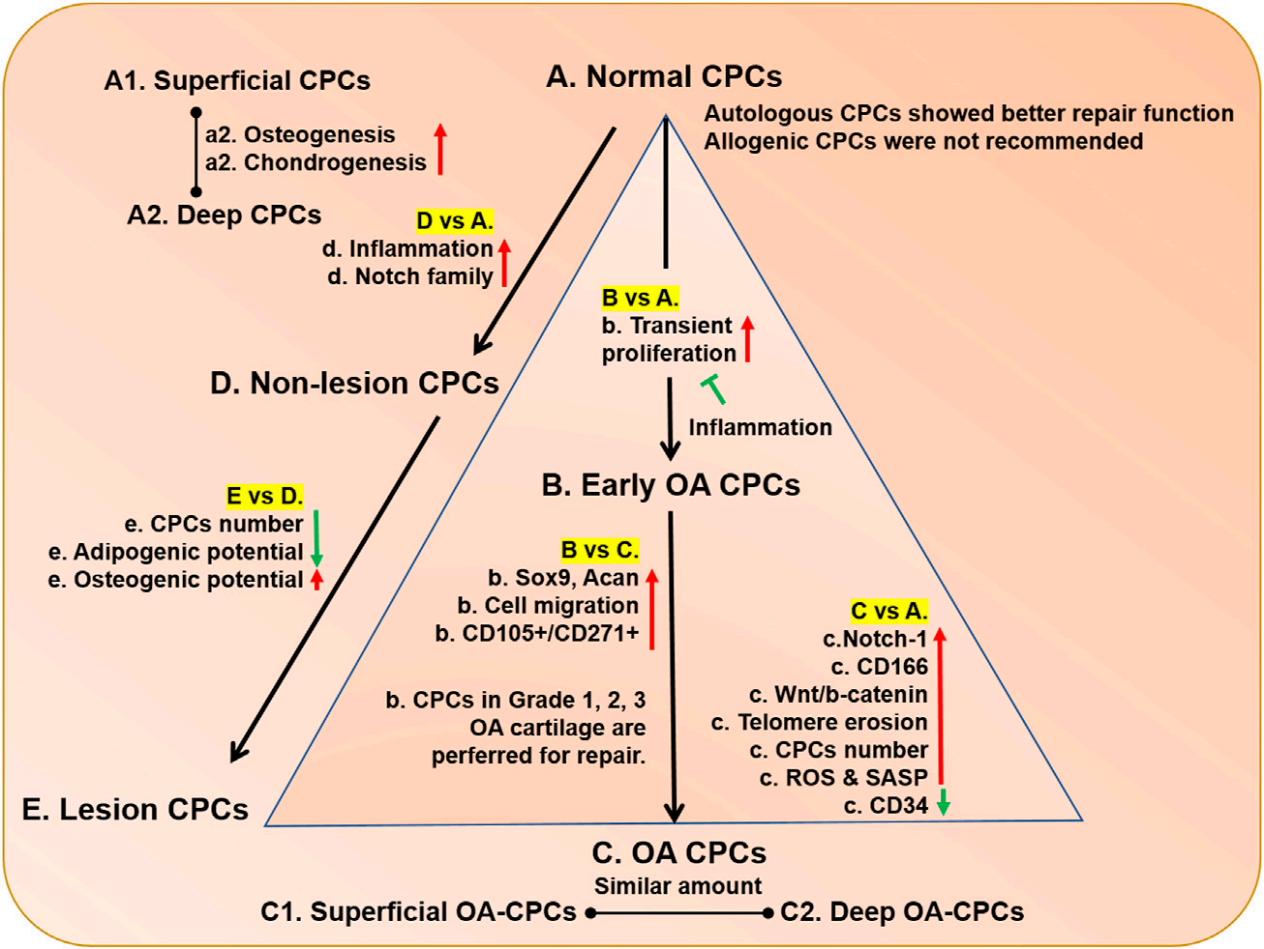
The comparison of CPCs in normal and OA cartilage. The CPCs in normal, early OA, and late OA cartilage were compared, while the CPCs in normal, non-lesion, and lesion OA cartilage were compared. The capitalized **(A–E)** means CPCs from healthy cartilage, early OA CPCs, OA CPCs, CPCs from non-lesion OA cartilage, CPCs from lesion OA cartilage, respectively. a, b, c, d, e, f, g in lowercase means the cell properties in A, B, C, D, E cells compared with cells being connected by line, respectively. The red arrow means upregulation; the green arrow means downregulation. The blue symbol means inhibition.

Autologous but not allogenic normal CPCs serves as an ideal cell resources for cartilage repair (Hiraoka et al., 2006), but the repairable process could be compromised during OA progression. As far as we know, OA is a chronic and low-level inflammatory disease, so it influenced CPCs persistently. Based on a comparative analysis of normal and OA cartilage, CD166^+^ and CD34^−^ were suggested as cell markers of CPCs (Vinod et al., 2020b). In addition to the overall markers, lots of cell markers were used in specific conditions. Normal CPCs, early OA CPCs and OA CPCs were often compared to investigate the alteration caused by OA progression (Table 3). The distribution, amount, and function of CPCs were investigated. Cell clusters are thought to be the histological hallmark of OA cartilage, which contains several cells with stem cell markers (Lotz et al., 2010), such as NOTCH 1 (Figures 4A vs. C) (Hiraoka et al., 2006). Notch family proteins and their receptors could not be detected in healthy human cartilage and were activated in the superficial zone and middle zone during OA progression (Grogan et al., 2009; Mahjoub et al., 2012). Some markers appeared, meanwhile some markers altered their localization. Prg4-positive cells were located in the superficial zone and shifted toward deeper zones during OA (De Luca et al., 2019). Based on colony-forming efficiency (CFE), CPCs increased 2-fold in OA cartilage compared with normal cartilage (Figures 4B vs. C) (Fellows et al., 2017). Cells from mild but not severe OA cartilage showed increased CPC markers (CD105 and CD166) after 2 weeks of culture *in vitro* (Mazor et al., 2017), which may reflect that the CPCs in early OA tried to repair the defect. Compared with normal CPCs, rat CPCs (rCPCs) only showed a transient proliferation increase, but it could not stop OA progression (Figures 4A vs. B) (Tong et al., 2015), which may be caused by the fewer CPCs in late OA (Mantripragada et al., 2018). Instead of fewer number of CPCs, the proliferation was also reduced in STR/Ort spontaneous OA mouse model (Zhang et al., 2019). Another reason for OA formation may be the changes of differentiation potentials. CD271^+^ cells were confirmed as multiple potential stromal cells that exhibited strong osteogenic differentiation capacity (Churchman et al., 2014), which increased during OA progression (Murphy et al., 2002). Furthermore, CPCs from higher grade (3–4) OA upregulated CD271 and decreased the chondrogenic potential (Figures 4B vs. C) (Wang et al., 2020b). In addition to cartilage, the number of mesenchymal progenitor cells in OA synovial fluid (SF) is also higher than that in normal (Jones et al., 2008) or rheumatoid arthritis (RA) SF (Jones et al., 2004).

**TABLE 3:** The comparison of CPCs in normal and OA cartilage.

Species	Age	Gender	Sites	Severity	Isolated methods	Results	Ref
Human	25–85 years old (N = 11)		Lateral tibial plateau	Normal cartilage	Fibronectin attachment	CPCs increased in OA cartilage but displayed increased senescent properties, such as telomere erosion	Fellows et al. (2017)
	54–85 years old (N = 11)		Lateral tibial plateau adjacent to cartilage lesions	OARSI grade 3.25 (mean) OA cartilage			
Human			Knee articular cartilage	Normal cartilage	A mixture of chondrocyte and CPCs	Notch-1^+^ cells increased in OA cartilage	Hiraoka et al. (2006)
				OA cartilage			
Human	22 ± 4 years (N = 3)		Knee articular cartilage	Normal cartilage	Fibronectin attachment	Lower CD34 and higher CD166 in OA CPCs	Vinod et al. (2020b)
	63 ± 7 years (N = 3)			Grade 4 OA cartilage			
Human	58–85 years old (N = 10)		Tibia plateau cartilage	OARSI grade 1–3 OA cartilage	A mixture of chondrocyte and CPCs	Cells from the mild OA cartilage showed higher CD105, Sox9 and Acan expression compared with severe OA cartilage after 14 days culture *in vitro*	Mazor et al. (2017)
Rat	12 weeks old		Hip and knee joints cartilage	Normal cartilage	Fibronectin attachment	CPCs showed a transient proliferative increase in early OA, but could be inhibited by inflammation	Tong et al. (2015)
	8-week-old			OA model	*In vivo*		
Human	45–76 years old (N = 15)	7 M/8 F	Load-bearing of lateral femoral condyle	Grade 1–2 OA cartilage	A mixture of chondrocyte and CPCs	Prevalence (CTPs/million cells) was not different between superficial and deep cartilage	Mantripragada et al. (2018)
Human	63.6 years (N = 28)	9 M/19 F	Lateral tibial plateau	Grade 1–2 OA cartilage	Migration	Stronger cell migration and more CD105+/CD271+ cells in higher grade OA CPCs	Wang et al. (2020b)
			Medial tibial plateau	Grade 3–4 OA cartilage			
Human	47–79 years old (N = 12)	4 M/8 F	Medial and lateral condyles cartilage	Normal looking and degraded OA cartilage	CD105+/CD166+ sorting	The CPCs number, proliferation, and adipogenic potential in lesion area decreased and osteogenic potential increased	Xia et al. (2016)

Instead of CPCs alteration during OA progression, CPCs from lesion area and non-lesion area of OA cartilage in same patient exhibited different properties. CD166^+^/CD105^+^ CPCs increased during OA progression in both nonlesion and lesion areas (Fickert et al., 2004). But compared with CPCs from nonlesion areas, those from lesion areas harbored stronger osteogenic and lower adipogenic potential with a reduction in cell percentage and proliferation rate (Figures 4D vs. E) (Xia et al., 2016). Non-lesion cartilage looks like normal cartilage, but the CPCs inside already changed with the up-regulation of inflammation (Figures 4D vs. A) (Xia et al., 2016).

Till now, CPCs from lower Outerbridge grade (grade 0–1) were recommended for cartilage repair (Xia et al., 2016). These data all indicated that CPCs increased their amount and were committed to restoring the cartilage defect. But for some reasons, the internal inflammation and external mechanical environment inhibited the regeneration process. A better understand of underlying mechanism of CPCs alteration would raise some new directions for OA therapy.

## The influence of external and inner factors on CPCs

### Injury

In our daily life, incidental injury happens to the knee during motion, torsion and compression. Degenerated cartilage and OA are rarely found in the young people. However, cartilage defects were commonly found in middle-aged people (Seol et al., 2014). When a sudden injury strikes the knee joint, the number of CD105^+^ cells increased and then migrated toward the injury site (Zhang et al., 2016) with the overexpression of IL8, CCL2, and VEGF (Wang et al., 2017; Zhou et al., 2014). Enzymatic treatment that mimics degeneration could accelerate CPC migration with gene upregulation of COL1 and COL2 (Seol et al., 2014). That is, CPC could respond to injury and start to repair the cartilage defect. But when the defects could not be repaired in a certain time, the proliferation and differentiation of CPCs are inhibited by the long-lasting inflammation in early OA and onset OA progression (Seol et al., 2014).

#### Inflammation

Inflammation is significantly harmful to cartilage, although it is essential for the onset of repair in several tissues, such as wound healing. Long-term and extended inflammation altered the properties of CPCs. IL-1β and its downstream factor nerve growth factor (NGF) are both negative for chondrogenesis and contribute to OA-CPC degeneration (Jiang et al., 2015). Inhibition of NF-κB could rescue chondrogenesis and proliferation of rCPCs, attenuating OA progression (Tong et al., 2015). MRL/MpJ “superhealer” mice (Table 4) could be protected from posttraumatic osteoarthritis (PTOA) because of the lower intra-articular and systemic inflammation, proving the association of inflammation and PTOA (Lewis et al., 2013). The CPC extracellular vesicles and miR221-3p derived from the mouse could contribute to cartilage repair (Wang et al., 2020a), which may implement their paracrine secretion function. Inflammation could not only inhibit CPCs proliferation, but also inhibit their extracellular matrix protein (Prg4) expression (Elsaid et al., 2014) that could prevent cartilage degeneration (Flannery et al., 2009). Therefore, chronic and extended inflammation may be a key factor to inhibit cartilage regeneration in middle-age and elder people.

**TABLE 4:** Mouse models have been used in CPCs investigation.

Mouse	Function	Results	Induced time	Examination time	Ref
Prg4-CreER (T2): Confetti	Prg4^+^ cells tracing	Superficial cells are self-renewing progenitors	at birth (TAM)	P5 to 6M	Kozhemyakina et al. (2015)
Col2-CreERt: Confetti	Col2^+^ cells tracing		E19.5 ± 1D (TAM)	P3 to 2M	
H2B (H2B)–GFP Tet-On	Slowly dividing cells labeling		E14.5-P2 (Dox)	P2, P18, and 1M	
Prg4^GFPCreERt2^; Rosa26^floxlacZ^	Prg4^+^ cells tracing	The progeny of Prg4^+^ at E17.5 are present in all articular cartilage. The progeny of Prg4^+^ at 1 month extend 2/3 the upper of the articular cartilage	E17.5/1M (TAM)	P0 to 12M/6 and 18M	Li et al. (2017)
Prg4^GFPCreERt2^; Rosa26^mTmG^	Prg4^+^ cells tracing				
Col11-CA-bcatER	β-catenin investigation in articular regions	SFZ thickness↑, Prg4↑, CPCs↑	2W (TAM)	5W	Yasuhara et al. (2011)
Col2CreER; β-catenin^fl/fl^	β-catenin investigation in articular regions	SFZ thickness↓, Prg4↓, CPCs↓	P5 to P7 (TAM)	7W	
CagCreER; β-catenin^fl/fl^	β-catenin investigation	Prg4↓, aggrecan↑, collagen 10↑	2W (TAM)	5W	
STR/Ort	Spontaneous OA model	CPCs exhibited lower proliferative and differentiation capacity (decreased CD44 and CD90)	-	4M/8M	Zhang et al. (2019)
MRL/MpJ	PTA resistance	MRL/MpJ mice showed lower inflammation and extracellular vesicles from MRL CPCs enhances articular cartilage repair	8W (DMM)	16W	Lewis et al. (2013), Wang et al. (2020a)

E, embryonic day; P, postnatal day; D, day; W, week; M, month; TAM, tamoxifen; Dox: doxycycline; DMM, destabilization of the medial meniscus.

### Aging

Instead of inflammation, there is a strong association between increasing age, chondrogenic potential and OA (Brophy et al., 2012). Cellular senescence played an important role in inhibiting pathological progression, such as cancer, but the accumulation of senescent cells during aging was harmful for tissue regeneration (He and Sharpless, 2017). Full depth chondrocytes could form cartilage pellets in SCID mouse, but passaged chondrocytes lose the capacity for pellet formation (Marcus et al., 2014). p16^INK4a^ is a marker of cell senescence that increased ∼50-fold in cartilage from 4 to 18-months mouse (Diekman et al., 2018; Jeon et al., 2017). Clearance of senescent cells (p16^INK4a+^ cells) could attenuate OA, but targeted deletion of the p16^INK4a^ gene in chondrocytes did not stop OA progression (Diekman et al., 2018; Jeon et al., 2017). In our pervious data, OA-derived CPCs showed senescence compared with non-OA CPCs and OA chondrocyte (Jacob et al., 2022), which may prove that OA progression may be induced by aged CPCs because of their strong inflammatory expression (Alsalameh et al., 2004). In addition to CPCs, p16^INK4a+^ muscle adult stem cells also failed to activate and expand, but entried into a full senescence state when injury happened (Sousa-Victor et al., 2014). Aged CPCs showed spontaneous osteogenic differentiation and lower chondrogenic differentiation (Chang et al., 2011). In our previously published data, sonic hedgehog, osteogenesis inducer, was highly expressed in OA-CPCs and could induce CPC senescence and chondrocyte apoptosis, which may be used as target and biomarkers for OA (Feng et al., 2021; Liu et al., 2022).

### Mechanical loading

**FIGURE 5:**
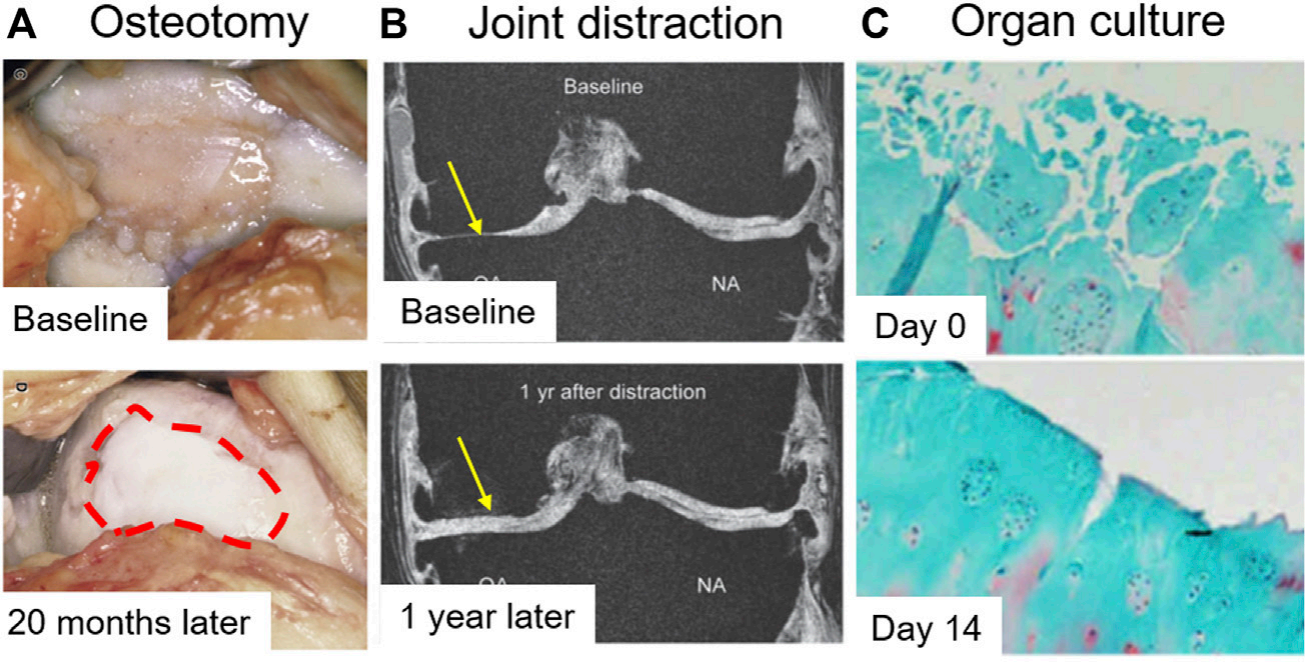
Evidence of cartilage rebuilding *in vitro* and *in vivo*. **(A)** The articular surface was observed 20 months after high tibial osteotomy (HTO). The red dot circle shows neocartilage formation (Intema et al., 2011). **(B)** Quantitative magnetic resonance imaging (MRI) of the joint was taken after 1 year of joint distraction (Koshino et al., 2003). **(C)** OA cartilage was cultured for 14 days. Safranin O-fast green staining was performed (Hoshiyama et al., 2015).

In OA patients, genu varus deformities were observed commonly with increased mechanical loading in the medial compartment, which induced asymmetric OA progression. Although cartilage was thought to be a non-self-healing tissue, joint offloading techniques by surgical intervention serve as a new approach for cartilage preservation and restoration. Realignment of the vaurs genum by high tibial osteotomy (HTO) (Figure 5A) and total joint distraction (Figure 5B), both can produce neocartilage that were confirmed by a second-look arthroscopic examination at 1 or 2 years after surgery (Intema et al., 2011; Koshino et al., 2003; Wiegant et al., 2013). The neocartilage was pure white and showed strong collagen II expression, while the minimum joint space width was significantly increased. The phenomena are appealing, but the underlying mechanism is still not clear. Recently, it is reported that overloading promotes chondrocyte senescence, and contributes to OA progression (Zhang et al., 2022). However, physiological mechanical stimulation could upregulate chondrogenic markers *in vitro* (Neumann et al., 2015), but nuclear factor kappa-B (NFκB) can abolish mechano-induced ECM synthesis (Luckgen et al., 2022). In addition, mechanical loading is also associated with aging that younger animals showed greater plasticity (Walsh et al., 2020). Therefore, that’s why the majority of patients are often elder varus/valgus deformities patients (Sharma et al., 2001), who suffered extended mechanical loading and cellular senescence.

### Nutrition and hypoxia

In addition to mechanical loading, nutrition supply was another key factor for cartilage homeostasis. OA cartilage clefts could be filled with extracellular matrix after 14 days of organ culture (Figure 5C), which may be induced by offloading conditions and abundant nutrition supply. Not ideally, the expression of GAG could not be reformed, which may be caused by limited incubation time (Hoshiyama et al., 2015). The medium contained high glucose with several growth facotrs, such as TGFβ, BMP, and IGF, which benefit cell chondrogenesis and proliferation. Platelet lysate is a natural growth factor-rich solution, which could be isolated from human blood and often be used in regenerative medicine. Compared with fetal bovine serum (FBS), human platelet lysate (hPL)-cultured CPCs showed increased proliferation, chondrogenic markers and osteogenic potential. The medium contained 14-fold upregulation of TGFβ1 (Kachroo et al., 2021). Moreover, platelet lysate could recruit CPCs, enhance the response to inflammatory signal (Carluccio et al., 2020), and upregulate hypoxia inducible factor-1 (HIF-1) (Nguyen et al., 2018), benefiting cartilage repair. Cartilage is an avascular tissue and the bioenergetic metabolism of chondrocyte is limited by the dense extracellular boundaries. Therefore, the hypothesis always is that physioxia and low glucose benefits cartilage homeostasis. In cartilage, chondrocytes are more sensitive to physioxia (5% O_2_) than CPCs (Anderson et al., 2018), but further physioxia (2% O_2_) benefit CPC chondrogenesis (Anderson et al., 2016). High glucose (25 mM) inhibited CPC colony-forming efficiency (CFE), but it increased GAG expression (Mantripragada et al., 2021), which is a key component of hyaline cartilage, so that we may choose different culture condition based on our purposes. Low-density and low-glucose condition *in vitro* was proved to enhance CPC proliferation, achieving CPC amplification for large knee cartilage defect (6–13 cm^2^) repair in humans (Jiang et al., 2016). Recently, a study proved that lipids but not glucose from blood repressed chondrogenesis of skeletal progenitor cells by inhibiting SOX9 (van Gastel et al., 2020), giving us a new direction for OA therapy based on comprehensive nutrition supplyment, including glucose, lipid, proteins, and O_2_.

## Discussion

Within this review, we aimed to evaluate the literatures about CPCs to address the question “why CPCs with strong self-renew and chondrogenic potential cannot repair the cartilage defect, resulting OA formation”. It shined insight into the reasons why CPCs lose their proliferation and chondrogenic differentiation abilities during development, maturation, aging and OA pathogenesis. Plenty of researches proved the chondrogenic potential with defined factors, including kartogenin (Liu et al., 2020a), BMP7 (most beneficial effects) (Riegger et al., 2018), BMP9 (less fibrillation) (Morgan et al., 2020), link protein N-terminal peptide (He et al., 2018), factor-rich platelet rich plasma (Vinod et al., 2019b), and bFGF (Shen et al., 2022). After a comprehensive comparison, including chondrogenic potential, telomere activity, and osteogenic/chondrogenic gene expression, migratory CPCs and clonal CPCs are more ideal for cartilage repair than enriched CPCs, dedifferentiated CPCs, BMSCs, and chondrocytes, which may due to their homogeneity. Furthermore, based on colony-forming efficiency and GAG content, CPCs were preferred for cartilage repair over those derived from synovium, infrapatellar fat pad (IPFP), bone marrow, and periosteum (Jessop et al., 2019; Mantripragada et al., 2019). Fibrin and thrombin solution with autologous CPCs could repair equine cartilage defects, proving the importance of scaffold and the preference of autograft (Frisbie et al., 2015). When cells were combined with platelet-rich plasma (PRP) scaffold for cartilage repair, CPCs showed superiority compared with BMSCs and chondrocytes (Zhang et al., 2019). But till now, few of them had been approved for clinical trials. BMSCs have been investigated for a longer time than CPC, which were thought as ideal “seed cells” for allogeneic transplant because of their low immunogenic potential, but the “immune privileged” property was denied recently (Ankrum et al., 2014). Moreover, a forty-six clinical studies showed that limited evidence is available regarding the clinical benefit of BMSCs for articular cartilage repair (Park et al., 2018). That is, autologous transplant or treated in local may be more ideal for clinical translation. However, the OA condition dramatically altered the properties of cells. BMSCs from OA patients exhibited reduced chondrogenic and adipogenic activity (Murphy et al., 2002). Surprisingly, chondrogenic induction of OA CPCs could activate OA hallmark markers (Hu et al., 2019), which may due to the lower expression of TGFBR1 (Liu et al., 2011). Activation mutation of TGFBR1 in mice showed neocartilage formation, which confirmed that TGFBR1 is more important for chondrogenesis than its ligand, TGFβ1 (Liu et al., 2011). Another TGFβ super-family member, BMP2 could induce chondrogenesis via Sox9 and osteogenesis via Runx2. BMP2 also could upregulate hypertrophy markers (Neumann et al., 2015), but the combination of BMP2 and soluble VEGFR1 (sVEGFR1) benefited skeletal stem cell chondrogenesis *in vivo* (Murphy et al., 2020), which revealed that the aginogenesis is negative for cartilage regeneration. Instead of grwoth factors, stabilization of heterochromatin by CLOCK promotes BMSC rejuvenation and cartilage regeneration (Liang et al., 2021), which may exhibit similar functions in CPCs. The results of cartilage repair are more important and pragmatic than chondrogenic potential alone. Therefore, how to achieve cartilage regeneration and restoration in clinical is the key point.

For autologous transplant, secondary damage is an issue, especially for cartilage. Autologous chondrocyte implantation (ACI) often isolated chondrocyte in nonbearing site and was used for cartilage defect, meanwhile the cartilage defect is an inducement for OA formation. Besides, the unchanged inner condition, especially in OA, may not maintain the repaired cartilage homeostasis, such as fibrosis after ACI surgery. Two reprogramming factors (c-Myc and Klf4) and one chondrogenic factor (SOX9) induce polygonal chondrogenic cells directly from adult dermal fibroblast cultures (Hiramatsu et al., 2011), which may be a new source of CPCs for cartilage repair. It may solve the lack number of local CPCs because of the easily accessible in other mature tissues than cartilage.

CPCs, as a type of cartilage-resident progenitor cell, showed multiple differentiation potential, including chondrogenic differentiation, which is the key demand for cartilage repair. The proliferation and chondrogenesis of CPCs were inhibited in OA cartilage, although CPC migration ability was retained. Moreover, spontaneous osteogenesis was found in OA CPCs, which may contribute to osteophyte formation. Therefore, how to rescue the proliferation and chondrogenic potential of CPCs locally is a vital event for OA attenuation and cartilage repair. The two abilities were significantly inhibited by inflammation and extended machinal loading. Offloading techniques was confirmed to repair cartilage lesions with neocartilage after one or 2 years of mechanical alteration, but the underlying mechanism was still unclear. The CPCs clusters in OA cartilage may be re-activated to form neocartilage when the extended mechanical stimuli dampened. New animal models are needed to investigate the origin of the neocartilage.

OA is a chronic inflammation disease, while lots of researches revealed the harmful function of inflammation in cartilage regeneration. Inflammation condition inhibited CPCs proliferation and chondrogenesis. Even in other diseases, such as infected wound defects, long-term and excessive inflammation could impair wound healing. An effective and sustained release anti-inflammation drug is required for inflammation control by one-time intra-articular injection. The biomaterial is a promising area to archive multiple functions that can hardly be archived in biology or medical areas (Tu et al., 2022). Based on the multiple etiology property (Vincent et al., 2022), combined functions in one single biomaterial, including mechanical support, anti-inflammation, growth factor binding, and nutrition supply, may be preferred for OA therapy than one single etiology targeted treatment.

OA usually remains asymptomatic until late, and reliable early markers for diagnosis are still lacking [123]. Cartilage defects had been ovserved in middle-aged people. Early markers are needed and can leave enough treat time for patients to manage OA course. At that time, the regeneration property of CPCs may not have been influenced by the harmful OA environment. Currently, several mouse models have been generated for OA research and cartilage repair (Table 4), which also can be used to investigate the origin of CPCs and the early markers in CPCs during OA.

In summary, CPCs showed strong regeneration ability in immature cells and visible proliferation and chondrogenic potential *in vitro* and could be used for cartilage repair after amplification culture and the combination of scaffolds. For OA patient, the internal and external environment is complicated, including aging, inflammation, mechanical stress. Clinical evidences of offloading technologies demonstrate that neocartilage could be regenerated under proper condition and the stem cells must be involved in the regeneration process. The local treatment to re-activate CPCs proliferation and to control inflammation release may tackle with these underlying etiologies and guiding OA therapy and upcoming clinical trials.

## Glossary

bCPCs: Bovine cartilage-derived progenitor cells
bFGF: Basic fibroblast growth factor
BMP2: Bone morphogenetic protein two
BMP7: Bone morphogenetic protein seven
BMP9: Bone morphogenetic protein 9
BMSC: Bone marrow mesenchymal stem cells
CCL2: C-C motif chemokine ligand two
CFE: Colony forming efficiency
CLOCK: Clock Circadian Regulator
COL1: Type I collagen
COL2: Type II collagen
CPCs: Cartilage-derived progenitor cells
CXCL12: C-X-C motif chemokine ligand 12
ECM: Extracellular matrix
FBS: Fetal bovine serum
FN: Fibronectin
HMGB1: High mobility group protein one
hPL: Human platelet lysate
IGF: Insulin like growth factor
IL-1β: Interleukin-1 beta
IL8: Interleukin-8
MRI: Magnetic resonance imaging
MSCs: Mesenchymal stem cells
NFκB: Nuclear factor kappa-B
NGF: Nerve growth factor
OA: Osteoarthritis
PDGF: Platelet derived growth factor
Prg4: Proteoglycan 4
RA: Rheumatoid arthritis
SF: Synovial fluid
SFZ: Superficial zone
TGFBR1: Transforming growth factor beta receptor one
TGFβ1: Transforming growth factor beta one
TNF-α: Tumor necrosis factor α
VEGF: Vascular endothelial growth factor
VEGFR1: Vascular endothelial growth factor receptor 1
TAM: Tamoxifen
Dox: Doxycycline
DMM: Destabilization of the medial meniscus.
